# Structure and Physical Properties of Ceramic Materials Based on ZrO_2_-Sc_2_O_3_ for SOFC Electrolytic Membranes Obtained from Powders of Melted Solid Solutions with a Similar Composition

**DOI:** 10.3390/membranes13080717

**Published:** 2023-08-01

**Authors:** Dmitrii Agarkov, Mikhail Borik, Ekaterina Buzaeva, Galina Korableva, Alexey Kulebyakin, Irina Kuritsyna, Nataliya Larina, Vladimir Kyashkin, Elena Lomonova, Filipp Milovich, Valentina Myzina, Polina Ryabochkina, Nataliya Tabachkova, Denis Zakharov

**Affiliations:** 1 Osipyan Institute of Solid State Physics RAS, Academician Osipyan Str., 2, 142432 Chernogolovka, Russia; eliseevagm@issp.ac.ru (G.K.); koneva@issp.ac.ru (I.K.); 2 Moscow Institute of Physics and Technology, Institusky Lane, 9, 141700 Doloprudny, Russia; 3 Prokhorov General Physics Institute of Russian Academy of Sciences, Vavilova Street, 38, 119991 Moscow, Russia; borik@lst.gpi.ru (M.B.); kulebyakin@lst.gpi.ru (A.K.); lomonova@lst.gpi.ru (E.L.); vamyzina@lst.gpi.ru (V.M.); ntabachkova@misis.ru (N.T.); 4 Institute of High Technologies and New Materials, National Research Ogarev Mordovia State University, Bolshevistskaya Street, 68, 430005 Saransk, Russia; katyabuzaeva@yandex.ru (E.B.); saharova.1996@mail.ru (N.L.); kyashkin@mail.ru (V.K.); ryabochkina@freemail.mrsu.ru (P.R.); 5 Department of Materials Science, Moscow Polytechnic University, Bolshaya Semyonovskaya Street, 38, 107023 Moscow, Russia; philippmilovich@gmail.com; 6 Department of Materials Science of Semiconductors and Dielectrics, National University of Science and Technology «MISIS», Leninskiy Prospect, 4, 119049 Moscow, Russia; deniszakharovm@mail.ru

**Keywords:** zirconia membranes, SOFC, single crystal, structure, conductivity

## Abstract

This paper presents the results of studying the phase composition, luminescent characteristics, and ionic conductivity of ceramic scandium-stabilized solid solutions of zirconium dioxide containing 9 and 10 mol% Sc_2_O_3_. Ceramic samples were prepared by sintering powders obtained by grinding melted solid solutions of the same composition. A comparative analysis of the obtained data with similar characteristics of single crystals has been carried out. Differences in the phase composition of ceramics and initial single crystals were found. The effect of the structure and properties of grain boundaries on the ionic conductivity of ceramic samples is discussed. It is shown that the differences in the ionic conductivity of ceramic samples and crystals are mainly due to changes in the structure and phase composition.

## 1. Introduction

Ceramic materials are widely used in energy storage devices and other fields [1,2,3]. The uniqueness of the physicochemical characteristics of solid solutions based on zirconium dioxide ensures their wide use for various practical applications [4,5]. Due to the presence of ionic conductivity at high temperatures, materials based on zirconium dioxide have become widespread as solid electrolytes used for the manufacture of oxygen-conducting membranes of various electrochemical devices, which include solid oxide fuel cells, gas sensors, oxygen partial pressure sensors, etc. [6,7]. The main requirements for an electrolyte to work efficiently are oxide ion conductivity must be sufficiently high (≈0.1 S/cm at operating temperature); low electronic conductivity; thermodynamic and chemical stability over a wide range of temperatures (from room temperature to 1000 °C); chemical inertness with respect to electrode materials; and reliable mechanical properties [8].

At present, a significant number of studies have been performed aimed at revealing the relationship between the structural features and ionic conductivity of solid solutions based on stabilized zirconia [9,10,11,12,13,14,15,16]. The cubic fluorite-type phase of scandia stabilized zirconia (ScSZ) shows the highest conductivity among all zirconia solid solutions [11]. SOFCs with electrolytic membranes based on ZrO_2_-Sc_2_O_3_ systems show the highest power characteristics compared to devices using membranes based on ZrO_2_-Y_2_O_3_. The use of electrolytic membranes from zirconium dioxide stabilized by scandium oxide makes it possible to reduce the operating temperature of the fuel cell, which improves the stability and reliability of electrochemical devices.

Currently, materials obtained by different ceramic technologies are used as electrolyte membranes for SOFCs. At the same time, it should be noted that the structure, ionic conductivity, and mechanical characteristics of ceramic materials significantly depend on the methods of obtaining ceramics, the type, purity, and fractional composition of the starting materials, temperature, and time conditions of synthesis (sintering).

An alternative way to obtain materials for electrolytic membranes is the growth of crystals [17,18,19,20]. Single crystals based on ZrO_2_-Sc_2_O_3_ solid solutions are good model objects, the study of which makes it possible to unambiguously reveal the influence of the phase composition and local structure on ionic conductivity, excluding the effects associated with the presence of grain boundaries.

The widespread use of ceramics in SOFC is due to the possibility of manufacturing electrolytic plates with dimensions of 10 × 10 cm [21,22] or more. Growing crystals of this size have no fundamental limitations [23]; however, it is associated with the solution of complex technical problems and does not seem to be economically feasible. Therefore, it is of particular interest to conduct comparative studies of the structural and transport properties of crystals and ceramic samples of the same compositions. To solve this problem, powders of precursors of ceramic materials were obtained by grinding melted solid solutions of similar composition, and ceramic samples were made. To study the local environment of cations in the crystal lattice by optical spectroscopy, Eu^3+^ ions were used as an optical probe [24,25]. For this, a small amount of Eu_2_O_3_ was introduced into the composition of the material.

The purpose of this work was to study the structure, spectral and luminescent characteristics and ionic conductivity of ceramic materials ZrO_2_-9 mol.% Sc_2_O_3_-0.1 mol.% Eu_2_O_3_, ZrO_2_-10 mol.% Sc_2_O_3_-0.1 mol.% Eu_2_O_3_ obtained from powders of melted solid solutions with similar composition.

## 2. Materials and Methods

Ceramic samples of the (ZrO_2_)_0.909_(Sc_2_O_3_)_0.09_(Eu_2_O_3_)_0.001_ and (ZrO_2_)_0.899_(Sc_2_O_3_)_0.10_(Eu_2_O_3_)_0.001_ compositions, designated as 9Sc0.1EuSZ and 10Sc0.1EuSZ in the text of the article, were obtained from powders made from crystals of similar compositions.

Crystals of 9Sc0.1EuSZ and 10Sc0.1EuSZ solid solutions were grown by directional melt crystallization at a rate of 10 mm/h in a water-cooled crucible 130 mm in diameter using direct high-frequency heating on a Kristall-407 setup (frequency 5.28 MHz, power 60 kW). Zirconium (ZrO_2_), scandium (Sc_2_O_3_), and europium (Eu_2_O_3_) oxides with a base oxide content of at least 99.96 wt.% were used as the raw materials.

**Figure 1:**
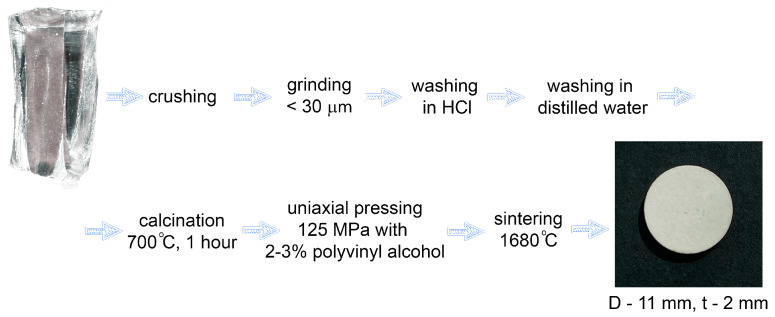
Diagram of the manufacturing routine of ceramics.

In the manufacture of powders, preliminary mechanical crushing of single crystals was carried out on a hydraulic press and subsequent grinding in a planetary mill. To clean the crushed powders from possible contaminants during crushing and grinding, they were treated with dilute hydrochloric acid, followed by washing with distilled water. After washing and filtering, the powders were calcined at a temperature of 700 °C in the air atmosphere for one hour. For the manufacture of ceramics, a powder with a particle size of less than 30 μm was used. The specific surface area of the 10Sc0.1EuSZ and 9Sc0.1EuSZ powders was ~4550 cm^2^/g and ~5280 cm/g, respectively. Ceramic samples were obtained by uniaxial pressing at a pressure of 125 MPa. A 2–3% solution of polyvinyl alcohol (PVA) was used as an organic binder during pressing. The samples were sintered in the air in a furnace with lanthanum chromite (LaCrO_3_) heaters in a zirconium oxide container (with a lid) placed in a magnesium–aluminum spinel crucible (with a lid) at a temperature of 1680 °C for 2 h; heating and cooling rates were 200 °C/h. Ceramic samples with a diameter of 11 mm and a height of 2 mm were made. Figure 1 shows a diagram of the manufacturing routine of ceramics.

The phase composition of powders and ceramics was studied by X-ray diffraction using an Empyrean diffractometer manufactured by PANNalitical D.V. (CuKα radiation, λ = 1.5414 Å) with a vertical type of goniometer and a PIXcel 3D detector, respectively. The diffraction patterns were interpreted using the JSPDS PDF 2 1911 database. The phase composition of the ceramics was also studied by Raman spectroscopy (RS) using a 633 nm laser as an excitation source.

Density was determined by hydrostatic weighing on a Sartorius hydrostatic weighing instrument.

The study of surface morphology and determination of the elemental composition of ceramics was carried out using scanning electron microscopy and energy-dispersive spectroscopy on a Quanta TM 3D 200i scanning electron microscope with a microanalysis system (EDS). SEM images were taken at an accelerating voltage of 20 kV in a high vacuum mode (~10^−3^ Pa).

The study of the spectral and luminescent properties was carried out by optical spectroscopy using Eu^3+^ ions as a spectroscopic probe. Luminescence spectra were recorded at T = 300 K using an inVia spectrometer manufactured by RENISHAW.

The electrical conductivity of the ceramic samples was studied in the 400–900 °C range with a Solartron SI1260 frequency analyzer in the 1 Hz–5 MHz region at a 24 mV AC current signal.

## 3. Results and Dictation

The obtained 9Sc0.1EuSZ ceramic samples had a density of ~5.09 g/cm^2^, the value of which was ~88% of the density of non-porous single crystals of the same composition (~5.78 g/cm^2^). The density of the 10Sc0.1EuSZ ceramic samples was ~4.97 g/cm^2^, which was ~86% of the density of single crystals of the same composition (~5.76 g/cm^2^).

**Figure 2:**
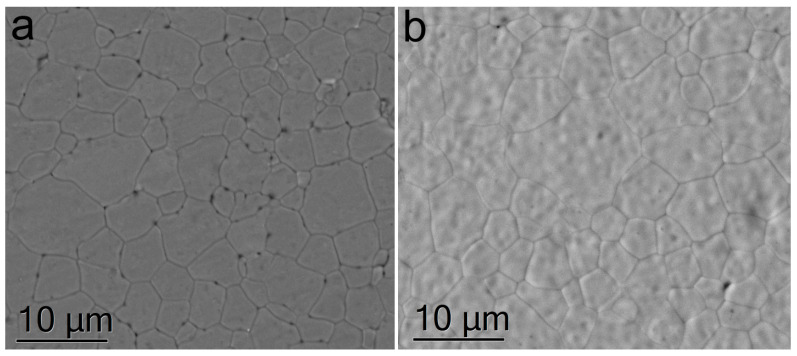
Microstructure of 9Sc0.1EuSZ (**a**) and 10Sc0.1EuSZ (**b**) ceramics.

Figure 2 shows the SEM image of the microstructure of the 9Sc0.1EuSZ and 10Sc0.1EuSZ ceramics.

The grain sizes in 9Sc0.1EuSZ and 10Sc0.1EuSZ ceramic samples practically did not differ and were in the range of 3–20 µm. The samples contained pores located mainly along the grain boundaries. EDS analysis did not detect the presence of impurities in the bulk of the grain and at the grain boundaries.

The phase composition of 9Sc0.1EuSZ and 10Sc0.1EuSZ crystals obtained by directional crystallization of the melt was studied earlier. Using X-ray diffraction, it was found that 9Sc0.1EuSZ crystals have a tetragonal structure, while 10Sc0.1EuSZ crystals are a mixture of cubic and rhombohedral phases [26].

**Figure 3:**
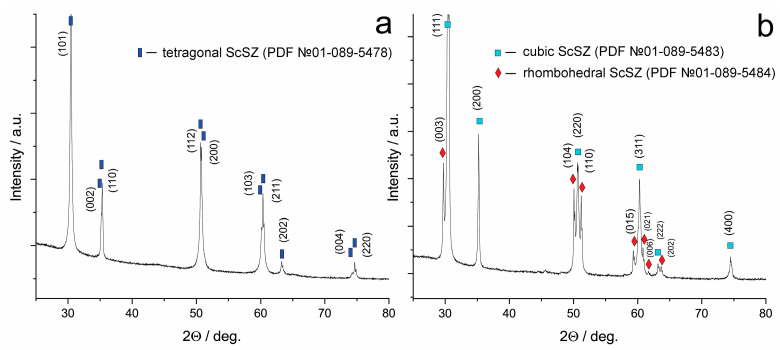
Diffractograms from 9Sc0.1EuSZ (**a**) and 10Sc0.1EuSZ (**b**) powders.

Figure 3 shows diffraction patterns from powder samples obtained by crushing and subsequent grinding crystals.

The diffraction pattern of the 9Sc0.1EuSZ powder contained only reflections characteristic of the tetragonal modification of zirconium dioxide. The diffraction pattern of the 10Sc0.1EuSZ powder contained diffraction maxima corresponding to the rhombohedral and cubic phases of solid solutions based on zirconium dioxide. Thus, it was detected that the mechanical grinding of crystals does not lead to a change in the phase composition.

**Figure 4:**
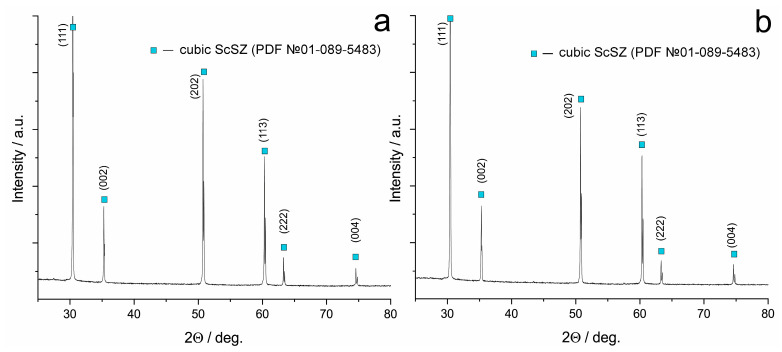
Diffractograms from 9Sc0.1EuSZ (**a**) and 10Sc0.1EuSZ (**b**) ceramic samples.

The phase composition of ceramic samples made from the obtained powders differs from the phase composition of the initial crystalline and powder samples. Ceramic 9Sc0.1EuSZ and 10Sc0.1EuSZ samples are single-phase and have a cubic fluorite-type structure (Figure 4).

The phase composition of ceramic and powder samples 9Sc0.1EuSZ and 10Sc0.1EuSZ was also studied by the method of Raman spectroscopy. For comparison, we also used Raman spectroscopy data obtained for crystals of these compositions [26].

**Figure 5:**
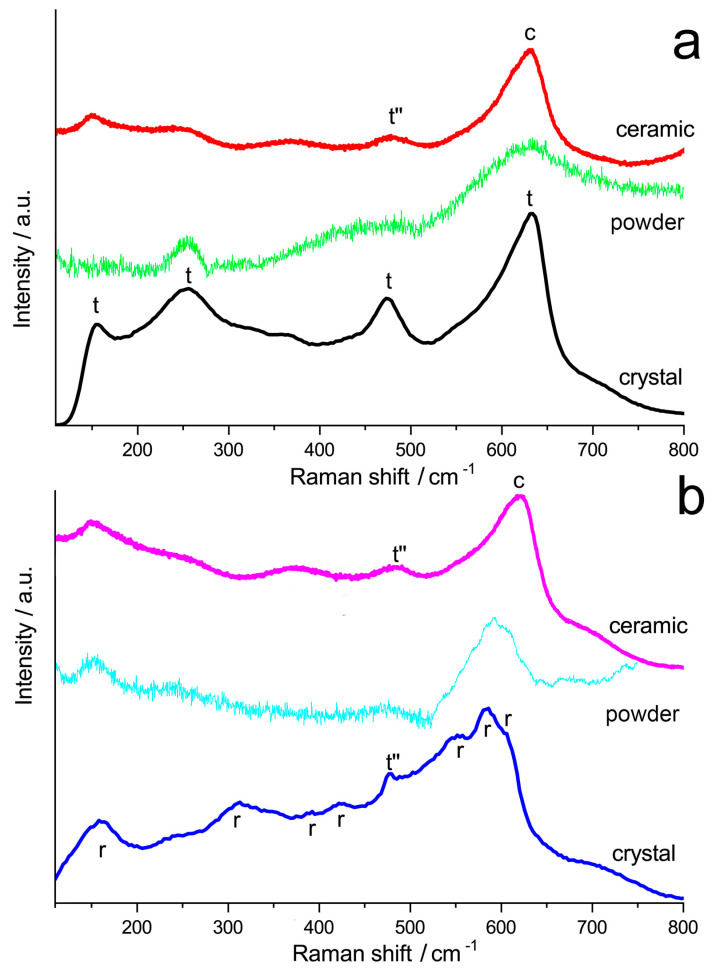
Raman spectra of 9Sc0.1EuSZ (**a**) and 10Sc0.1EuSZ (**b**) samples: peak designation ‘t’ is for tetragonal phase, ‘t”’ is for t”-phase and ‘r’ is for rhombohedral phase, ‘c’ is for cubic phase.

Figure 5 shows the Raman spectra of crystals, powders, and ceramic samples 9Sc0.1EuSZ and 10Sc0.1EuSZ. The spectrum of the 9Sc0.1EuSZ crystal and powder contain bands (154, 254, 474, 632 cm^–1^) characteristic of the tetragonal phase [27]. The bands are significantly broadened, which may be due to the presence of a second phase, possibly cubic. The spectrum of the 9Sc0.1EuSZ ceramic sample contains bands (150, 365, 477, 630 cm^–1^), which are more characteristic of the pseudocubic structure of the t″ phase [28]. The greatest changes occurred in the Raman spectra of 10Sc0.1EuSZ ceramic samples as compared with the spectra of crystals or powder of the same composition. The Raman spectrum of 10Sc0.1EuSZ crystals corresponds to the rhombohedral structure (160, 243, 311, 351, 393, 424, 454, 495, 551, 584, 605 cm^−1^) [29]. The bands of the spectrum are broadened, and, in addition, the spectrum contains a band at 478 cm^−1^ attributed to the t″-phase. The comparison of the Raman spectra of powders and crystals showed that the grinding of crystals does not cause changes in the phase composition of the samples, which fully corresponds to the data of X-ray phase analysis. The Raman spectrum of the 10Sc0.1EuSZ ceramic sample is similar to the spectrum of the 9Sc0.1EuSZ ceramic sample. Thus, despite the different phase composition of the initial powders 9Sc0.1EuSZ and 10Sc0.1EuSZ used for the manufacture of ceramics, single-phase ceramic samples with a t″-phase structure were obtained. This may be due to the high temperature (1680 °C) of heat treatment during the sintering of ceramic samples and the cooling rate (200 °C/h), which makes it possible to preserve the high-temperature cubic phase. When growing single crystals by directional crystallization of the melt, the cooling of the grown crystals from the melting temperature to room temperature takes a longer time, depending on the volume of the crystallized melt [30]. It was established [26] that, despite the uniform distribution of the components of the solid solution along the length of the 9Sc0.1EuSZ and 10Sc0.1EuSZ crystals, the phase composition and local structure, which reflects the distribution of oxygen vacancies relative to the cations of the solid solution, is inhomogeneous along the length of the crystal, which is associated with different cooling conditions of single crystals in an ingot of a crystallized melt.

**Figure 6:**
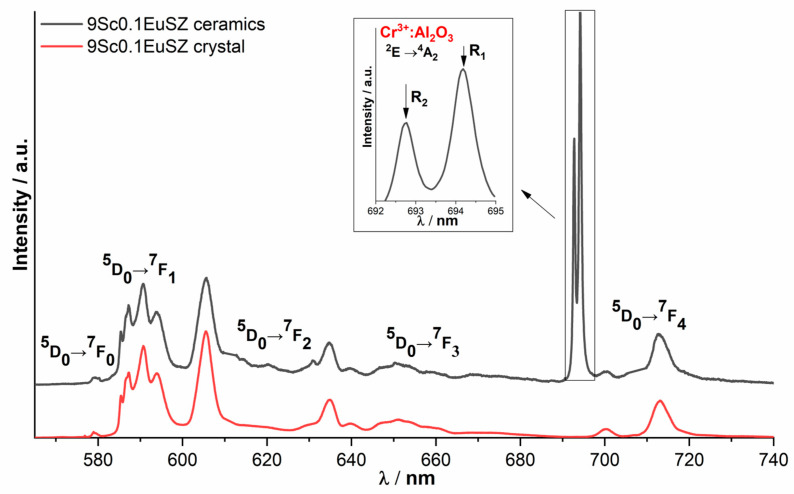
Luminescence spectra of 9Sc0.1EuSZ ceramics and single crystal, λ_ex_ = 532 nm, T = 300 K; inset shows luminescence lines of Cr^3+^ ions in Al_2_O_3_ for the ^2^E → ^4^A_2_ transition.

The ionic conductivity of materials based on zirconium dioxide strongly depends on the position of oxygen vacancies in the crystal lattice. The local environment of cations can be used to measure optical spectroscopy. Figure 6 shows the luminescence spectra of the 9Sc0.1EuSZ ceramics and the single crystal, recorded upon excitation to the ^5^D_1_ level by radiation with a wavelength of 532 nm at T = 300 K, due to the optical transitions ^5^D_0_→^7^F_0_, ^5^D_0_→^7^F_1_, ^5^D_0_→^7^F_2_, ^5^D_0_→^7^F_3_, ^5^D_0_→^7^F_4_ of Eu^3+^ ions.

The contour shape of the luminescence spectra of Eu^3+^ ions of solid solutions of 9Sc0.1EuSZ and 10Sc0.1EuSZ ceramics, characterized by a cubic structure, is close to the shape of the contour of a 9Sc0.1EuSZ single crystal with a tetragonal structure, which is due to the characteristic set of optical centers of Eu^3+^ ions in cubic and tetragonal crystals based on dioxide zirconium. These include Eu^3+^ ions seven coordinated with respect to oxygen, and also Eu^3+^ ions in which there is no oxygen vacancy in the first coordination sphere, but it is present in the second one, and Eu^3+^ ions with oxygen vacancies are present in the far coordination spheres.

**Figure 7:**
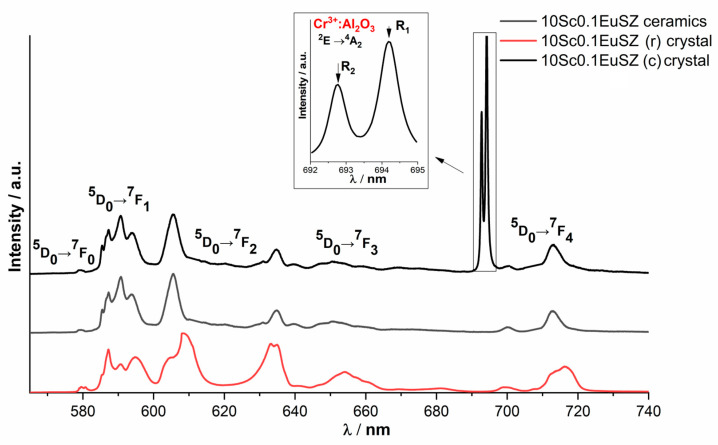
Luminescence spectra of 10Sc0.1EuSZ single crystals and ceramics, λ_ex_ = 532 nm, T = 300 K.

The luminescence spectrum of Eu^3+^ ions for the 10Sc0.1EuSZ solid solution recorded in different parts of the crystal differs, since it is a mixture of cubic and rhombohedral phases. The luminescence spectra of Eu^3+^ ions for 10Sc0.1EuSZ ceramics recorded in different parts of the ceramic do not differ in contour shape and correspond to the contour shape of the 10Sc0.1EuSZ luminescence spectrum with a cubic structure (Figure 7).

A distinctive feature of the luminescence spectra of 9Sc0.1EuSZ and 10Sc0.1EuSZ ceramics is the presence of two intense narrow lines in the region of 693 and 694.5 nm, which are absent in single crystals and do not belong to optical transitions of Eu^3+^ ions.

It should be noted that similar lines in the luminescence spectra were detected by us earlier when studying the luminescence spectra of Eu^3+^ ions in ceramics (ZrO_2_)_0.909_(Y_2_O_3_)_0.09_(Eu_2_O_3_)_0.001_ obtained from powders of ground crystals [31].

Our earlier analysis of the literature data [32] revealed that the intense narrow lines in the region of 692.8 and 694.2 nm are due to the optical transition ^2^E → ^4^A_2_ in the luminescence spectrum of Cr^3+^:Al_2_O_3_. Based on this, we concluded that the ceramic samples obtained in this work contained Cr^3+^:Al_2_O_3_ as an uncontrolled impurity.

Probably, the origin of the chromium impurity is associated with the use of LaCrO_3_ heaters during their heat treatment in insufficiently tightly closed crucibles made of aluminum-magnesium spinel (MgAl_2_O_4_). Since ceramic samples were sintered at a high temperature (1680 °C), chromium ions could evaporate from LaCrO_3_ heaters. The volatility of chromium in LaCrO_3_ at high temperatures is a well-known problem [33]. It is assumed that evaporation occurs according to the reaction:

2LaCrO_3_ ↔ La_2_O_3_ + Cr_2_O_3_ (1)

Chromium oxide at temperatures above 1200 °C can also be reduced to metallic chromium [34]. According to the literature data, the solubility of Cr_2_O_3_ in Al_2_O_3_ is unlimited [35], and since the recorded luminescence spectra contain only lines characteristic of Cr^3+^ ions in Al_2_O_3_, it can be concluded that Cr_2_O_3_ predominantly interacts with Al_2_O_3_, forming a solid solution Cr_2_O_3_-Al_2_O_3_.

**Figure 8:**
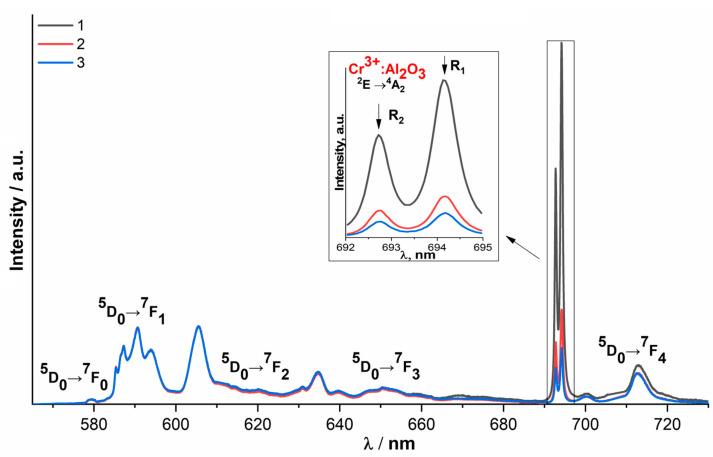
Luminescence spectra recorded from different regions of 9Sc0.1EuSZ ceramics, λ_ex_ = 532 nm, T = 300 K.

The change in the intensities of the luminescence lines of Cr^3+^ ions in Al_2_O_3_ relative to the luminescence lines of Eu^3+^ ions in different regions of the ceramic surface (Figure 8) indicates that the uncontrolled impurity is unevenly distributed in ceramic samples.

**Figure 9:**
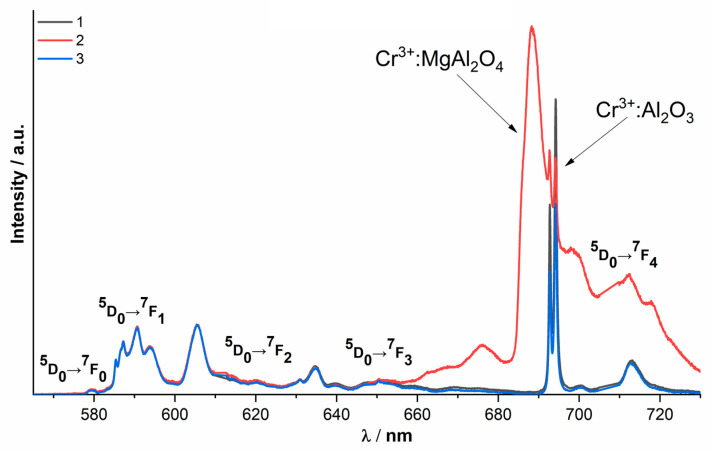
Luminescence spectra recorded from different regions of ceramics 10Sc0.1EuSZ, λ_ex_ = 532 nm, T = 300 K.

It should be noted that when recording the luminescence spectra from different regions of the 10Sc0.1EuSZ ceramic sample, along with the Cr^3+^:Al_2_O_3_ impurity, another uncontrolled impurity was detected, corresponding to the Cr^3+^:MgAl_2_O_4_ spinel (Figure 9).

An analysis of the published data revealed that the luminescence spectrum of Cr^3+^:MgAl_2_O_4_ contains a broad line in the region of 687 nm due to the transition of the Cr^3+^ ion from the excited state ^2^E_g_ (^2^G) to the ground state ^4^A_2g_ [36].

The presence of this uncontrolled impurity is also associated with the technological process. It is likely that the origin of the Cr^3+^:MgAl_2_O_4_ impurity is associated with heat treatment in a crucible made of aluminum-magnesium spinel (MgAl_2_O_4_).

**Figure 10:**
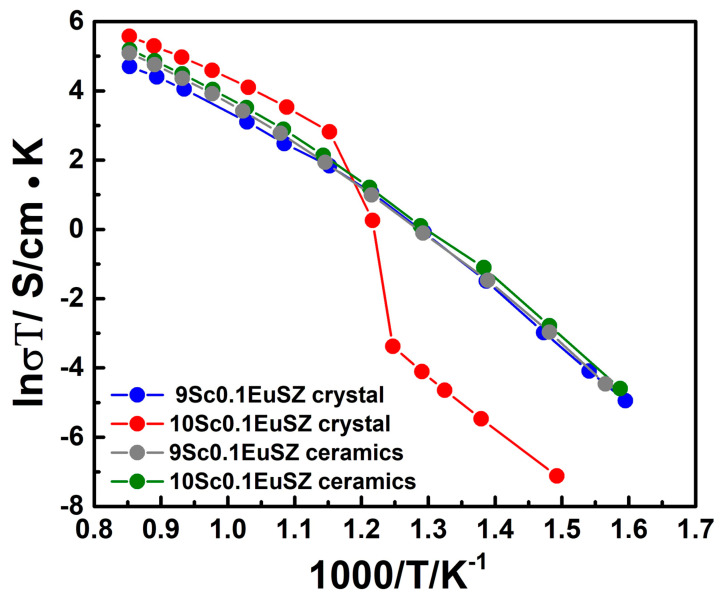
Temperature dependence of the conductivity of the studied ceramic samples and initial crystals 10Sc0.1EuSZ and 9Sc0.1EuSZ.

Figure 10 shows the temperature dependencies of the specific conductivity of the studied ceramic samples and initial crystals of 9Sc0.1EuSZ and 10Sc0.1EuSZ solid solutions in Arrhenius coordinates.

The temperature dependence of the conductivity of 10Sc0.1EuSZ crystals exhibits a jump in conductivity associated with the phase transition from the rhombohedral to the cubic phase. The specific electrical conductivity for the 10Sc0.1EuSZ crystal in the high-temperature region (973–1173 K) exceeds the values for the 9Sc0.1EuSZ crystals and is 0.225 and 0.095 S/cm at a temperature of 1173 K, respectively.

The value of specific electrical conductivity for ceramic samples 10Sc0.1EuSZ is slightly lower than that of the initial crystals in the temperature range of 973–1173 K, and it is 0.153 S/cm at 1173 K. There is no jump in the conductivity on the temperature dependence of the conductivity of the 10Sc0.1EuSZ ceramic sample. In the temperature range of 480–550 °C, corresponding to the temperature interval of the phase transition of the cubic phase into the rhombohedral one, the conductivity of ceramics 10Sc0.1EuSZ exceeds the conductivity of crystals. When the temperature drops from 550 °C to 480 °C, the conductivity of crystals drops sharply from 5.7 × 10^−2^ S/cm to 4 × 10^−5^ S/cm, and for ceramics, the conductivity decreases from 5.7 × 10^−2^ to 1.6 × 10^−3^ S/cm. In the temperature range below 480 °C, a significant difference in the conductivity of crystals and ceramic samples remains.

The value of specific electrical conductivity for ceramic samples 9Sc0.1EuSZ is slightly higher than that of the initial crystals in the temperature range of 973–1173 K, and it is 0.139 S/cm at a temperature of 1173 K. The high conductivity is apparently related to the structural differences between ceramics and crystals. Ceramic samples have the structure of a pseudocubic t″-phase, and the initial crystals are tetragonal.

The observed changes in the conductivity of crystals and ceramic samples are probably due to a combination of various factors, in particular, the structure and properties of grain boundaries. It is known that highly symmetrical phases (cubic fluorite and pseudocubic t″) of scandium-stabilized zirconium dioxide solid solutions have the highest oxygen ionic conductivity. The conductivity of the tetragonal modifications is noticeably lower, while the conductivity of the rhombohedral phases is the lowest [37]. At the same time, the electrical properties of grain boundaries in ceramic materials strongly depend on the type and concentration of impurities segregated in the region of grain boundaries [38]. The conductivity of 9Sc0.1EuSZ ceramics over the entire temperature range is higher than the conductivity of crystals of the same composition, since ceramics have a pseudocubic t″ structure, in contrast to the tetragonal structure of crystals. Probably, in this case, grain boundary effects make an insignificant contribution to the total ionic conductivity. The conductivity of 10Sc0.1EuSZ ceramics containing the pseudocubic t″ phase in the low-temperature region exceeds the conductivity of two-phase (cubic + rhombohedral) crystals. In the high-temperature region, the phase composition of ceramics and crystals does not differ. The observed lower conductivity of ceramic samples can be associated with an increased electrical resistance at the grain boundaries.

## 4. Conclusions

Ceramic samples 9Sc0.1EuSZ and 10Sc0.1EuSZ were obtained by uniaxial pressing at 125 MPa followed by powder sintering at 1680 °C for 2 h. We used initial powders with a particle size of no more than 30 μm, obtained by grinding preliminarily synthesized single crystals of similar composition. Differences in the phase composition of ceramics and initial powders are found. For example, the 9Sc0.1EuSZ and 10Sc0.1EuSZ ceramic samples were single-phase with a pseudocubic t″ phase structure. At the same time, the crystals and initial 9Sc0.1EuSZ powders had a tetragonal structure, while the 10Sc0.1EuSZ powders were a mixture of cubic and rhombohedral phases. 

The spectral and luminescent characteristics of 9Sc0.1EuSZ and 10Sc0.1EuSZ ceramic samples did not reveal significant differences in the local structure with respect to single crystals. Optical spectroscopy revealed the presence of uncontrolled impurities Cr^3+^:Al_2_O_3_ and Cr^3+^:MgAl_2_O_4_ in all ceramic samples.

A study of the ionic conductivity of ceramic samples in the temperature range of 400–900 °C showed that the conductivity of 9Sc0.1EuSZ ceramics over the entire temperature range, and 10Sc0.1EuSZ in the low-temperature region, is higher than the conductivity of crystals of the same composition. An analysis of the obtained data on the ionic conductivity of ceramics and crystals shows a decisive contribution to the total ionic conductivity of the crystal structure. The observed lower conductivity of 10Sc0.1EuSZ ceramic samples in the high-temperature region is probably due to the presence of uncontrolled impurities and increased electrical resistance at the grain boundaries.

Thus, from the point of view of practical application, the use of powders made from crystals as initial precursors makes it possible to obtain ceramic solid electrolytes by a relatively simple method of solid-phase sintering. However, it is necessary to exclude contamination by uncontrolled impurities during the production of ceramics.

## Data Availability

All the data are available within the manuscript.
